# TfR1 binding with H-ferritin nanocarrier achieves prognostic diagnosis and enhances the therapeutic efficacy in clinical gastric cancer

**DOI:** 10.1038/s41419-020-2272-z

**Published:** 2020-02-05

**Authors:** Xiaojing Cheng, Kelong Fan, Lin Wang, Xiangji Ying, Andrew J. Sanders, Ting Guo, Xiaofang Xing, Meng Zhou, Hong Du, Ying Hu, Huirong Ding, Ziyu Li, Xianzi Wen, Wenguo Jiang, Xiyun Yan, Jiafu Ji

**Affiliations:** 1 Key Laboratory Carcinogenesis and Translational Research (Ministry of Education/Beijing), Division of Gastrointestinal Cancer Translational Research Laboratory, Peking University Cancer Hospital & Institute, Beijing, China; 2 Key Laboratory of Protein and Peptide Pharmaceutical, Chinese Academy of Sciences and University of Chinese Academy of Sciences, Beijing, China; 3 Key Laboratory Carcinogenesis and Translational Research (Ministry of Education/Beijing), Department of Gastrointestinal Surgery, Peking University Cancer Hospital & Institute, Beijing, China; 4 Department of Gastrointestinal Surgery, Beijing Shijitan Hospital, Capital Medical University, Beijing, China; 5 Cardiff China Medical Research Collaborative (CCMRC), Cardiff University School of Medicine, Heath Park, Cardiff, UK; 6 Key Laboratory Carcinogenesis and Translational Research (Ministry of Education/Beijing) Department of Biobank, Peking University Cancer Hospital & Institute, Beijing, China; 7 Key Laboratory Carcinogenesis and Translational Research (Ministry of Education/Beijing), Division of Central Laboratory, Peking University Cancer Hospital & Institute, Beijing, China

**Keywords:** Targeted therapies, Cancer stem cells

## Abstract

H-ferritin (HFn) nanocarrier is emerging as a promising theranostic platform for tumor diagnosis and therapy, which can specifically target tumor cells via binding transferrin receptor 1 (TfR1). This led us to investigate the therapeutic function of TfR1 in GC. The clinical significance of TfR1 was assessed in 178 GC tissues by using a magneto-HFn nanoparticle-based immunohistochemistry method. The therapeutic effects of doxorubicin-loaded HFn nanocarriers (HFn-Dox) were evaluated on TfR1-positive GC patient-derived xenograft (GC-PDX) models. The biological function of TfR1 was investigated through in vitro and in vivo assays. TfR1 was upregulated (73.03%) in GC tissues, and reversely correlated with patient outcome. TfR1-negative sorted cells exhibited tumor-initiating features, which enhanced tumor formation and migration/invasion, whereas TfR1-positive sorted cells showed significant proliferation ability. Knockout of TfR1 in GC cells also enhanced cell invasion. TfR1-deficient cells displayed immune escape by upregulating *PD-L1*, *CXCL9*, and *CXCL10*, when disposed with IFN-γ. Western blot results demonstrated that TfR1-knockout GC cells upregulated Akt and STAT3 signaling. Moreover, in TfR1-positive GC-PDX models, the HFn-Dox group significantly inhibited tumor growth, and increased mouse survival, compared with that of free-Dox group. TfR1 could be a potential prognostic and therapeutic biomarker for GC: (i) TfR1 reversely correlated with patient outcome, and its negative cells possessed tumor-aggressive features; (ii) TfR1-positive cells can be killed by HFn drug nanocarrier. Given the heterogeneity of GC, HFn drug nanocarrier combined with other therapies toward TfR1-negative cells (such as small molecules or immunotherapy) will be a new option for GC treatment.

## Introduction

Gastric cancer (GC) is one of the most common types of cancer and leading causes of cancer-related death worldwide^1^. Nearly half of diagnosed GC is in China annually around the world^2^. Chemotherapy is one of the most commonly used methods for pre- or postoperation of the GC treatment, and this therapy strategy has been demonstrated to provide survival benefit or decrease lymph node metastasis to patients^3–5^. However, chemotherapy drugs will bring serious side effects to patients by their indiscriminate drug distribution and severe toxicity. Although many efforts have been made to investigate the molecular mechanisms of GC, few clearly targeted drugs are developed to treat GC patients in the clinic. It has been reported that targeted HER2 (human epidermal growth factor receptor 2) and VEGFR2 (vascular endothelial growth factor receptor 2) inhibitor drugs could prolong the survival of some GC patients^6,7^. However, this part of the population accounts for only about 20% of GC patients. There is more space for GC to dig targeted drugs or methods for improving the treatment.

To overcome this challenge, nanocarriers give such an advantage to carry drugs to preferentially loci, thus increasing drug aggregation and reducing side effects^8–10^. Previous study indicated that H-ferritin (HFn) as a nanocarrier can specifically bind to transferrin receptor 1 (TfR1 or CD71, in human), which guarantees iron supply by binding of iron-loaded transferrin and ferritin, even like a gate for pathogen selection to enter cells^11–14^. Mammalian ferritin is a natural spherical iron-storage protein, and two types of ferritin exist, namely, heavy-chain ferritin (HFn) and light-chain ferritin (L-ferritin, LFn)^13,15^. However, only HFn has been shown to target human malignant cells through its receptor TfR1^13,16^. Although the receptor of LFn in mice has been confirmed as Scara5^17,18^, its receptor in human has not been clearly elucidated. Recent studies indicated that HFn nanocarriers can encapsulate iron oxide nanoparticles (magnetoferritn, M-HFn) and load drugs by TfR1-mediated specific binding without extra modification^10,12^. It can be used in diagnostic and targeted therapy in tumor, and even transverse the blood–brain barrier to kill glioma tumor cells, with reduced toxicity and side effects and high tolerated dose^13^.

Previous study indicated that doxorubicin-loaded HFn nanocarrier (HFn-Dox) has a ten-fold higher drug accumulation, lower drug exposure of normal organs, and more efficiently cleared from the body compared with free Dox in mouse treatment model^10^. Therefore, in this study, we first investigated the clinical role of TfR1 on GC. We found that TfR1 was highly expressed in GC cells, and reversely correlated with the patient poor survival. TfR1-negative sorted GC cells showed tumor-initiating features. Knockout of TfR1 by CRISPR displayed immune escape when treated by IFN-γ, and activated Akt and STAT3-signaling pathways. Then for measuring the therapeutic value of HFn-Dox on TfR1-positive cells, we examined the effect of HFn-Dox in TfR1-positive GC patient-derived xenograft (GC-PDX) models. Taken together, our data suggest that TfR1 might be a potential identification biomarker in GC-targeted therapy. As the malignant and heterogeneity nature of tumor, combination therapy might achieve a better treatment effect: one with HFn drug nanocarrier system targeted to TfR1-positive cells; the other with inhibitors antagonistic to TfR1-negative cells.

## Results

### Correlation of TfR1 with clinicopathological parameters

**Fig. 1:**
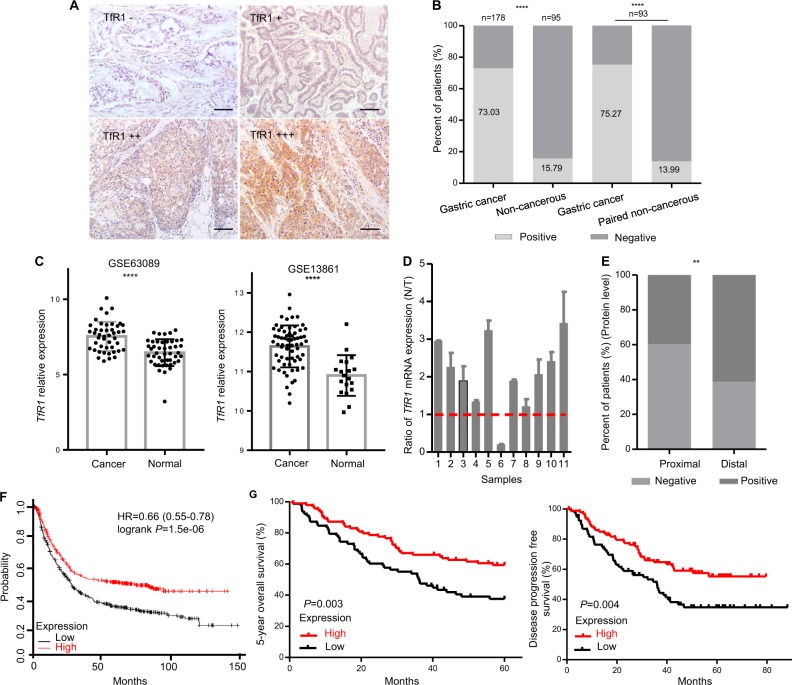
TfR1 protein expression in GC patients reversely correlated with poor prognosis. **a** Different staining scores with M-HFn nanoparticles detecting TfR1 in GC tissues by IHC, scale bars: 50 µm. **b** Expression level of TfR1 protein in GC and their (or matched) adjacent noncancerous tissues. **c** *TfR1* mRNA expression was significantly upregulated in GC tissues compared with adjacent normal mucosa in GES63089 and 13861 from GEO datasheets, respectively. **d** Ratio (T/N) of TfR1 mRNA expression in 11 paired primary GC patients, which was determined by qPCR (lower panel). Their expression levels were normalized by an internal control (*GAPDH*). **e** IHC staining that indicated high TfR1 protein expression was significantly associated with location of GC. **f** Kaplan–Meier survival analysis of OS obtained from public gene expression datasets. **g** Kaplan–Meier analysis of 5-year survival with low vs. high TfR1 protein expression status (left); Kaplan–Meier survival analysis of DFS with low vs. high TfR1 protein expression status (right). ^*^P < 0.05; ^**^P < 0.01; ^****^P < 0.0001.

TfR1 expression was evaluated by using a magneto-HFn (M-HFn) nanoparticle-based immunohistochemistry (IHC) method in GC and adjacent noncancerous mucosa tissues. TfR1 was expressed highly in tumor cells (Fig. 1a). We also validated the consistency of tumor detection with the traditional IHC, with an anti-TfR1 antibody in GC tissues (Fig. S1). TfR1 was positive in 130 of 178 tumor tissues, and 15 of 95 in the noncancerous mucosa tissues (73.03% vs. 15.79%, *P* < 0.0001), respectively. In paired tissues, the TfR1 expression was 75.27% vs. 13.99% (GC vs. paired noncancerous tissues, *P* < 0.0001). (Fig. 1b). The level of *TfR1* mRNA in normal and GC specimens was analyzed by the Gene Expression Omnibus (GEO) database (GES13861 and GES63089), which indicated that *TfR1* mRNA level was significantly higher in GC tissues compared with adjacent noncancerous mucosa tissues (*P* < 0.0001 and *P* < 0.0001, respectively) (Fig. 1c). We measured the expression level of *TfR1* mRNA in 11 pairs of primary GC tissues, and matched adjacent noncancerous mucosa tissues. Consistent with these results, a relatively higher expression of *TfR1* was found in GC tissues compared with its matched adjacent noncancerous mucosa tissues (Fig. 1d).

Among the TfR1 protein levels in GC patients, a higher proportion of tumors located at the distal region was TfR1 positive than that of tumors in the proximal region (61.2%, 79/129 vs. 39.6%, 19/48, *P* = 0.010, Fig. 1e). However, there was no significant correlation with age, gender, lymph node metastasis, depth of invasion, distant metastasis, and differentiation, gross type, tumor size, Lauren, and tumor-node-metastasis (TNM) stage of GC patients. Detailed data are shown in Table S1.

### TfR1 expression reversely correlated with poor prognosis in primary GC

Overall GC patient survival in *TfR1* mRNA level was analzyed by Kaplan–Meier method, using the online tool (http://kmplot.com/analysis), showed that a high level of *TfR1* expression was significantly associated with a better overall survival (OS) in GC patients (Fig. 1f). Similar results were detected in our data based on protein levels of TfR1 (*P* = 0.003 (OS) and 0.004 (PFS, progression-free survival), respectively; Fig. 1g). For the protein level of TfR1, the median 5-year OS time was 34.89 ± 2.42 months for TfR1-low patients and 44.15 ± 2.11 months for TfR1-high patients, respectively, and the median PFS time was 31.68 ± 2.57 vs. 37.66 ± 2.22 months, respectively.

The univariate Cox’s model for 5-year survival of GC patients revealed that TfR1 expression was one of the prognostic factors (hazard ratio (HR) = 0.533; 95% confidence interval (CI): 0.348–0.817; *P* = 0.004), and the other prognostic factors included lymph node metastasis (*P* < 0.001), depth of invasion (*P* < 0.001), distant metastasis (*P* < 0.001), and tumor size (*P* < 0.001). Using the multivariate Cox’s model, TfR1 was shown to be a novel independent prognostic factor of 5-year OS (HR = 0.450; 95% CI: 0.286–0.708; *P* = 0.001), compared with other independent prognostic factors including lymph node metastasis (*P* < 0.001), depth of invasion (*P* = 0.016), distant metastasis (*P* < 0.001), and tumor size (*P* = 0.017). Detailed data are shown in Table 1.

**Table 1:** Univariate and multivariate Cox’s models for 5-year overall survival of gastric cancer patients

Variables	Gastric cancer univariate analysis			Gastric cancer multivariate analysis		
	HR	95% CI	*P* value	HR	95% CI	*P* value
Age			0.883			0.113
≤60 vs. >60	1.032	0.676–1.577		1.452	0.915–2.305	
Gender			0.295			0.109
Male vs. female	0.782	0.493–1.240		0.670	0.410–1.094	
Lymph node metastasis			**<0.001**			**<0.001**
No vs. N1 + 2 + 3	18.465	5.825–58.531		11.947	3.726–38.311	
Depth of invasion			**<0.001**			**0.016**
T1 + 2 vs. T2 + 3	16.146	3.969–65.690		5.815	1.388–24.366	
Distant metastasis			**<0.001**			**<0.001**
M0 vs. M1	7.084	3.972–12.632		7.711	3.957–15.026	
Differentiation			0.298			
Poor vs. moderate + good	0.797	0.520–1.222				
Gross type			0.161			
Ulcerative type vs. others	0.636	0.337–1.198				
Tumor size			**<0.001**			**0.017**
≤5.0 cm vs. >5.0 cm	2.624	1.700–4.050		1.720	1.101–2.688	
Location			0.122			
Distant vs. proximal	0.700	0.446–1.100				
Lauren						
Diffuse vs. intestinal	1.395	0.753–2.583	0.290			
Diffuse vs. mixed	0.934	0.534–1.635	0.811			
TfR1			**0.004**			**0.001**
Low vs. high	0.533	0.348–0.817		0.450	0.286–0.708	

*Proximal* cardiac and gastroesophageal junction, *distal* gastric.

### HFn-encapsulated Dox showed superior antitumor effects on GC-PDX tumor

**Fig. 2:**
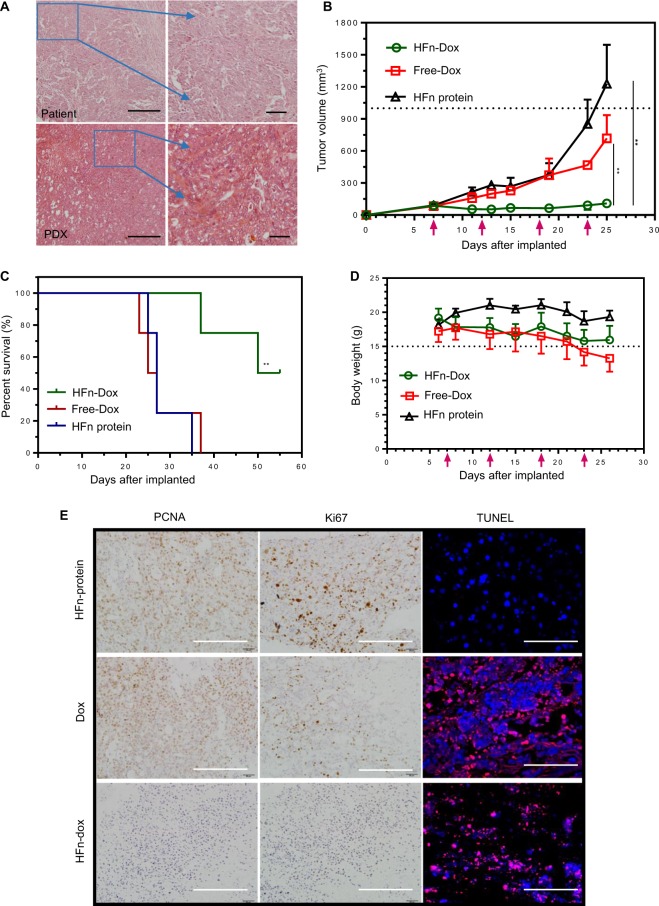
HFn-encapsulated Dox superiorly killed GC-PDX tumors. **a** H&E staining showed the similar histology of the patients and their corresponding PDX-derived tumors. **b** Tumor growth curves for different mouse groups were indicated. Red arrows: dose injection for every 5 days. **c** Mouse survival curves in different groups with Kaplan–Meier analysis. **d** Body weight changes in GC-PDX model. Red arrows: dose injection every 5 days. **e** Detection of proliferative and apoptotic cells in different groups after drug treatment. Scale bars, (**e** left two panels) 100 µm; (**e** right panel) 50 µm.

For the therapy effects of HFn nanocarriers encapsulating Dox, we selected TfR1-positive GC-PDX models treated with Dox-loaded HFn. The size-exclusion chromatogram of HFn-Dox and unloaded HFn is shown in Fig. S2. PDX models maintain the same genetic characteristics (methylation status, mutations, and resistance to therapy) observed in the patient from whom they were derived^19,20^. Hematoxylin–eosin (HE) staining showed the similarity of histological features between the patient tissue and its derived ones (Fig. 2a). HFn-Dox group significantly inhibited the tumor growth compared with free-Dox and HFn groups (108.99 ± 4.05 mm^3^ vs. 717.66 ± 218.00 mm^3^ and 1229.61 ± 365.05 mm^3^), presenting the tumor growth inhibition (TGI) rate of 91.1% for HFn-Dox compared with that of 41.6% for free Dox (*P* < 0.01) (Fig. 2b). There was also a significant improvement of survival for the HFn-Dox group compared with free-Dox and HFn groups (55 days vs. 26 and 27 days, both *P* < 0.01) (Fig. 2c), and with less weight loss in the HFn-Dox group compared with that of other two groups (Fig. 2d). Cardiotoxicity is a major limiting factor in clinical Dox-based therapy. Our work thus adds evidence that HFn-Dox treatment can substantially decrease in Dox-associated cardiomyopathy. This was also shown in a recent study that injections of HFn-Dox up to 20 mg/kg doses did not result in significant cardiotoxicity^10^.

Considering the drug effect in tumor tissues, we conducted histological analysis by using two proliferation markers: PCNA and Ki67, and TUNEL staining for cell apoptosis detection (Fig. 2e). There were less proliferation and more extensive apoptosis in the HFn-Dox group compared with free-Dox and HFn protein group, demonstrating that HFn-Dox possesses an obviously killing role in GC cells.

### TfR1-negative expression cells possessed tumor-initiating properties through in vitro and in vivo assays

**Fig. 3:**
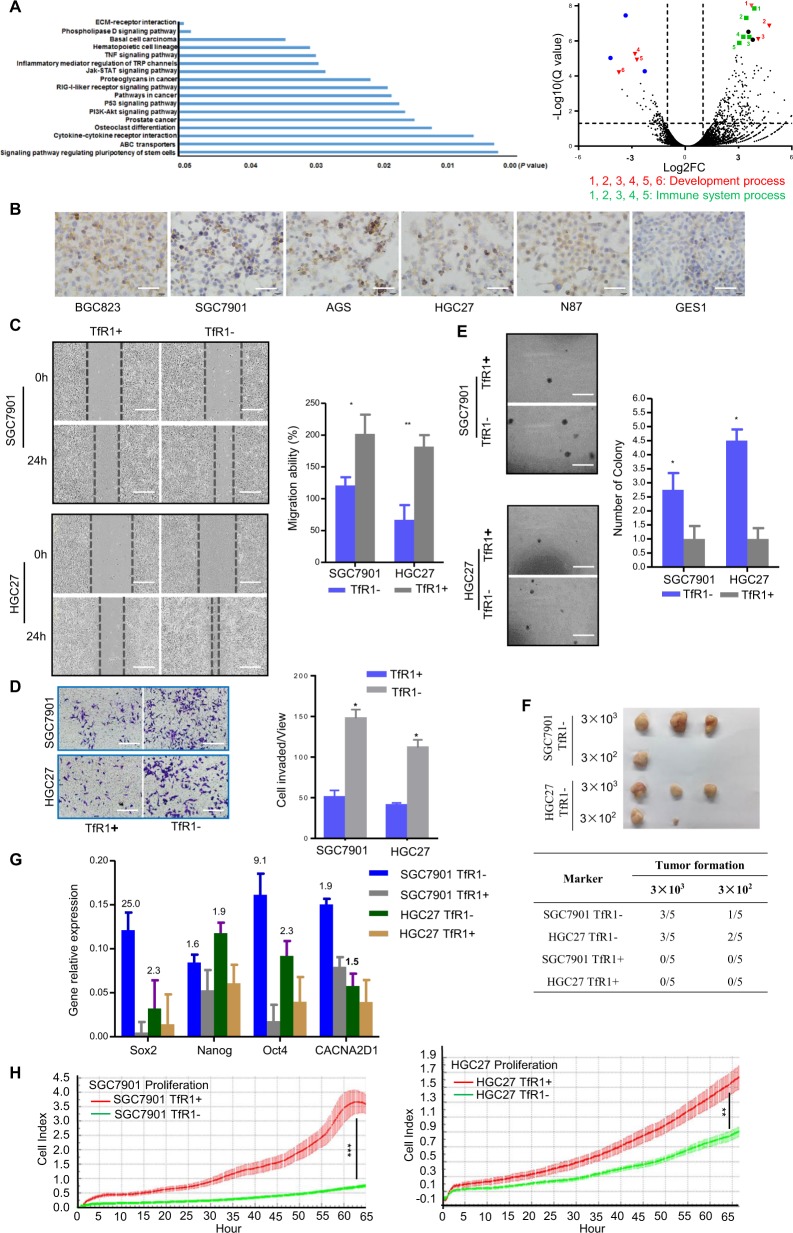
GC cells with the absence of TfR1 possess tumor-initiating like properties through in vitro and in vivo assays. **a** RNA-seq profiles for sorted TfR1-negative and -positive cells were analyzed. Significant signaling pathway (left panel) and volcano plot illustrated the differentially expressed genes between TfR1-negative and -positive cells (right panel, fold change > 2.0 or <2.0; *Q* value < 0.05). Blue, green, and red colors indicated various genes belonging to different groups of cell processes. **b** TfR1 was overexpressed in six GC cells (BGC823, SGC7901, AGS, HGC27, N87, and GES1). **c**–**e** Absence of TfR1 promoted cell migration, invasion, and colonogenicity by wound-healing assay, Boyden chamber invasion assay, and colony formation assay. Scale bar: 100 µm. **f** Analysis of TfR1 sorted ± cell tumorigenity following transplantation with different numbers of cells into NOD/SCID mice. **g** *Sox2*, *Nanog*, *Oct4*, and *CACNA2D1* mRNA relative expression was determined by qPCR. Their expression levels were normalized by an internal control (*GAPDH*). **h** TfR1-positive sorted cells showed obviously cell proliferation ability detected by real-time RTCA instrument. *P < 0.05; **P < 0.01, ***P < 0.001.

Given TfR1 clinical results and the tumor heterogeneity, we further investigated the function of TfR1 differentially expressed cells. We analyzed expression profiles within SGC7901 TfR1− and TfR1+ sorted cells using RNA-seq (Fig. 3a). Significant signaling pathway and volcano plot illustrated the differentially expressed genes between TfR1− and TfR1+ sorted cells (fold change > 2.0 or <2.0; *Q* value < 0.05), which mainly focused on molecules participating in pluripotency of stem cells, drug resistance, and cytokine–cytokine receptor interaction (Table S2).

TfR1 was overexpressed in six GC cells (BGC823, SGC7901, AGS, HGC27, N87, and GES1) (Fig. 3b). TfR1-negative sorted cells promoted the cell migration and invasion in HGC27 and SGC7901 cells by wound healing and Boyden chamber invasion assays (Fig. 3c, d), respectively. TfR1− sorted cells displayed a higher colony-forming efficiency compared with their respective counterpart TfR1+ sorted cells (Fig. 3e). Then to confirm the malignant property of these two sorted TfR1 populations, we compared the tumorigenic ability of TfR1− and + sorted cells. We found that 3000 TfR1− sorted cells formed tumors in three out of five subjects in both HGC27 and SGC7901 cells, and even fewer cells (300) were still able to form tumors. Nevertheless, there was no tumor formation with the corresponding number of TfR1+ sorted cells during the observed days (Fig. 3f). Furthermore, qPCR results indicated that the expression level of *Sox2*, *Nanog*, *Oct4*, and *CACNA2D1* mRNA was higher in TfR1− sorted cells compared with TfR1+ ones (Fig. 3g). However, TfR1− sorted cells showed significantly lower cell proliferation ability compared with TfR1+ sorted cells (Fig. 3h). These results demonstrate that GC cells with the absence of TfR1 possess tumor-initiating properties.

**Fig. 4:**
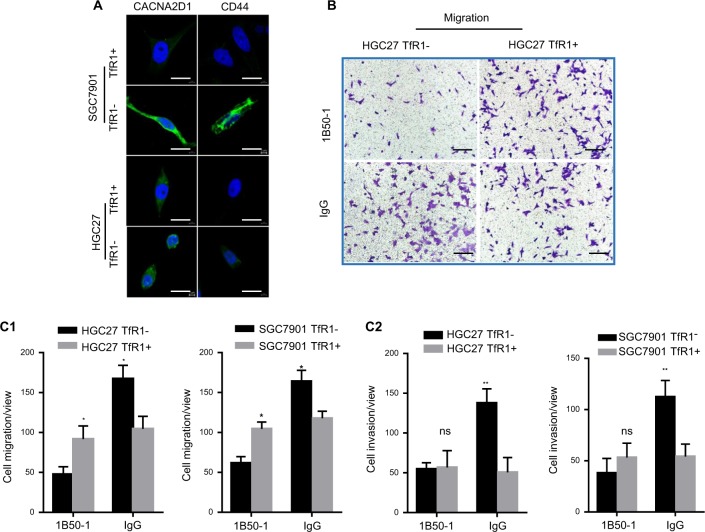
Migration/invasion of TfR1-negative cells was inhibited by the antibody toward CACNA2D1. **a** IF analysis indicated TfR1-sorted negative and positive cells co-expressing CACNA2D1 and CD44. **b** TfR1-sorted negative and positive cells were assayed for their migration/invasion abilities, with 17 μg/ml 1B50-1, and their matched mouse IgG antibody, using a Boyden chamber assay. Bars represent the mean and SD for three independent experiments. Scale bars, 10 µm; ^*^P < 0.05; ^**^P < 0.01. Scale bars, 2.5 µm. SD standard deviation.

As TfR1− sorted cells had progenitor cell properties, we selected the calcium channel α2δ1 subunit (CACNA2D1) as a target for inhibiting the movements of TfR1− sorted cells, which is one of the tumor-initiating molecules (TIMs) found in recurrent hepatocellular carcinoma^21^. First, we examined CACNA2D1 and CD44 markers in both sorted TfR1 cells using immunofluorescence (IF). TfR1− sorted cells expressed higher levels of these molecules (Fig. 4a). Furthermore, we selected 1B50-1, a monoclonal antibody targeting CACNA2D1, to explore treatment effects against TfR1− sorted cells. As shown in Fig. 4b, c, IB50-1 showed a significant suppressive effect on the migration and invasion of the TfR1− sorted cells, purified from HGC27 and SGC7901 cells.

### TfR1-knockout cells enhanced responsiveness to IFN-γ treatment

**Fig. 5:**
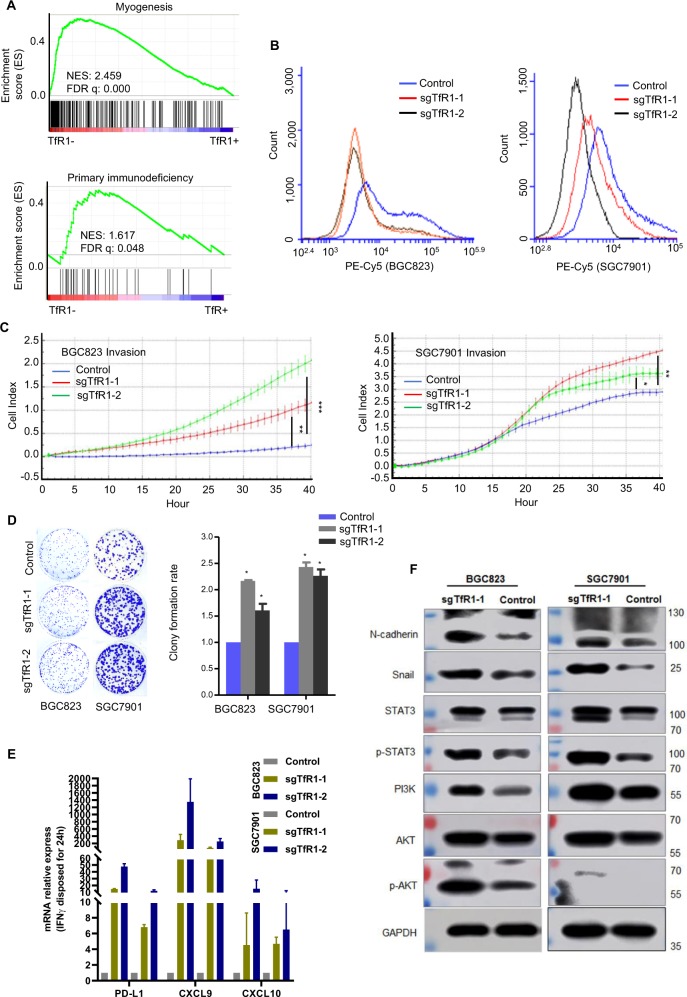
TfR1-knockout cells displayed malignant properties and responded to IFN-γ treatment. **a** GSEA plot based on RNA-seq (upper panel) or TCGA data (lower panel) indicated TfR1-negative cells correlated with developmental and immune deficiency. **b** TfR1-knockout cells were identified by flow cytometry analysis. **c** TfR1-knockout GC cells showed high invasion ability evaluated by RTCA real-time analysis instrument. **d** Knockout of TfR1 increased cell colonogenicity. **e** *PD-L1*, *CXCL9*, and *CXCL10* mRNA level was enhanced in TfR1-knockout cells stimulated with IFN-γ (100 ng/ml) for 24 h. **f** The correlation of EMT- related markers, STAT3/AKT signaling with TfR1, were detected by Western blot. NES normalized enrichment score. ^*^P < 0.05; ^**^P < 0.01; ^***^P < 0.001.

GSEA with TCGA STAD data on hallmark or KEGG gene sets indicated that myogenesis and primary immunodeficiency-related gene signatures were positively correlated in *TfR1* low-expressed cells (Fig. 5a). To investigate the function of TfR1 in GC cells, we selected TfR1-knockout cells with CRISPR–Cas9, and confirmed its expression with flow cytometry analysis (Fig. 5b). To further investigate the function of TfR1 in GC cells, knockout of TfR1 in BGC823 and SGC7901 cells markedly promoted cell invasion and clonogenicity by Matrigel invasion and colony formation assays (Fig. 5c, d). As primary immunodeficiency-related genes were correlated with *TfR1* low-expressed cells, we detected fold changes of *PD-L1*, *CXCL9*, and *CXCL10* mRNA levels in TfR1-knockout cells, which were enhanced after IFN-γ treatment (Fig. 5e). Recent studies demonstrated that interferon pathway may improve the immune checkpoint blockade therapy^22^. But the correlation of TfR1-negative cells and the immune checkpoint blockade therapy needs to be further elucidated. Moreover, as CD44 is used as a marker for cells with stem-like characteristics^23,24^, stratification of patient groups based on double expression of CD44+ and TfR1− improved the single predictive value of CD44 in patients with OS and PFS (Fig. S3a). Knockout of TfR1 in GC cells promoted the expression of mesenchymal markers (N-cadherin and Snail), and activated AKT and STAT3 signaling, as shown by Western blot (Fig. 5f).

### Analysis of TfR1-related tumor–genome features based on TCGA datasets

We performed gene set enrichment analysis (on Hallmark gene sets) to identify major biological processes using the Cancer Genome Atlas (TCGA) RNA-seq datasets (quartile highest and lowest *TfR1* expression samples) (Fig. S3b). KEGG gene set analysis revealed that vascular smooth muscle contraction, calcium-signaling pathway, and primary immunodeficiency significantly correspond to *TfR1* low-expression cases (Fig. S3b1). However, cell cycle, DNA replication, and RNA degradation (Fig. S3b2) correspond to *TfR1* high-expression cases. In addition, we found that *CD8A* and *CD8B* were reversely correlated with *TfR1* (*TFRC)* expression in TCGA RNA-seq datasets (Fig. S3c). Low *TfR1* mRNA levels were associated with a substantial worse survival in GC patients with high *CD8* mRNA levels divided by the mean of *CD8* (*CD8A* + *CD8B*)/2, but not in the low *CD8* mRNA group (Wilcoxon *P* = 0.0489 and 0.4298, respectively) (Fig. S3d).

## Discussion

This study was the first to demonstrate the clinical value of TfR1 based on HFn nanoparticle in GC as a potential prognostic indicator and therapeutic target. We used HFn nanoparticle to assess the clinical value of TfR1 in GC, and found that its expression is reversely associated with poor prognosis of GC patients, independent of their clinicopathological characteristics. Employing HFn as a nanocarrier (binding with TfR1 in the cell membrane), we first demonstrated that HFn-Dox can significantly improve the treatment efficacy of Dox to the GC-PDX model. Within the treatment cycle, the HFn-Dox almost completely inhibited the growth of tumor, and HFn-Dox treatment significantly increased the survival time and OS rate of tumor-bearing mice. As tumor cells are a highly heterogeneous population, we further testified that TfR1-negative GC cells possessed the tumor-initiating cells (TIC) properties and immune escape features through fluorescence-activated cell sorting (FACS) or CRISPR knockout through in vitro and in vivo assays.

IHC assays or mRNA expression analyzed with GEO datasets indicated that TfR1 expression in GC tissues was significantly higher than its expression in adjacent noncancerous mucosa tissues. Just as conventional antibody-based immunohistochemical assays for cancer detection, this novel M-HFn nanoparticle-based method identifying TfR1-positive cells can also distinguish tumors from normal tissues^12^. Previous studies demonstrated that transferrin (Tf) is an essential component in cell growth and iron-requiring metabolic processes^25,26^; thus, TfR1 is more highly expressed on rapidly growing cells such as on tumor cells^27,28^.

Although this is the first study on GC, the findings on the expression pattern here bear similarities to previous reports that TfR1 can be endogenously overexpressed in a variety of cancers, including lung^29,30^, colon^31^, pancreas^32^, and breast^33^. It is interesting to note the finding that TfR1 high expression significantly correlated with a favorable OS of GC patients. This phenomenon contrasts with that seen with breast cancer, in which TfR1 has been indicated as a poor marker for prognosis predicting breast cancer patients who respond to tamoxifen^34^. However, Ohkuma et al.^35^ reported that gastric adenosquamous cancer cells with the absence of TfR1 (CD71) possess tumor-initiating cell properties. This phenomenon might explain from the side that GC cells with TfR1 low expression have malignant features. Furthermore, several previous studies reported TfR1 deficiency in stem cells, including malignant leukemic^36,37^, keratinocyte^38^, and hematopoietic stem cells^39^.

Analysis of tumor–genome features (GSEA on KEGG and hallmark gene sets) based on RNA-seq and TCGA datasets revealed that the upregulated genes were enriched in signaling pathways regulating the pluripotency of stem cells, cytokine–cytokine receptor interaction, myogenesis, and primary immunodeficiency, which was significantly enriched in *TFRC* low-expression cells or groups. *PD-L1*, *CXCL9*, and *CXCL10* mRNA expression level was detected through fold changes (the highest changes are 100-fold) in TfR1 low cells compared with its high cells when stimulated by IFN-γ. These might be hinted for us that TfR1 low cells possessed the resistance of antitumor immune response^40,41^. However, the correlation of TfR1-negative cells and the immunotherapy needs to be further elucidated. As TfR1-negative cells displayed stem cell-like features, we found that 1B50-1 targeting CACINA2D1 could substantially inhibit TfR1-negative cell movement using Boyden chamber assay. These results guided us to choose a combination of this different class of agents (HFn chemicals, inhibitors for TIMs, and immune checkpoint blockade) for clinical application. Just like the ongoing clinical trial, chemotherapy, radiotherapy, and targeted therapy combine with PD1 therapy^42^. Combination therapy represents an effective strategy in treatment of cancer.

Our work provides an alternative approach for GC-targeted therapy as another form of chemotherapy drugs: (1) HFn nanocarriers with drugs could specifically kill GC cells, which were TfR1 positive in a receptor-dependent manner, and dozen-fold higher than their corresponding normal cells^43^; (2) HFn (drug) nanocarriers could passively target GC by their unique diameter size (12 nm), which is an ideal size for enhanced permeability and retention effects of the vasculature (EPR effect); (3) based on the feature of TfR1-negative cells, selecting other antagonists (antibody or small molecules toward tumor-initiating molecules) or immunotherapy as a combination with HFn drug nanocarriers might be an ideal treatment method for GC. A further validation of the combination treatment needs to be explored in GC-PDX models.

This study has indicated that HFn-Dox nanocarriers would be a beneficial choice for patients with GC in this context, but one marker alone will not truly reveal the malignant nature of cancer tissues; combination antagonist to other inhibitors for TIMs or immunotherapy with HFn drug nanocarrier systems might achieve a better treatment effect. However, our current studies demonstrated that TfR1 is a potential prognostic biomarker, and HFn drug nanocarrier makes an ideal therapeutic approach by targeting TfR1 in GC cells.

## Materials and methods

### GC patients and specimens

In total, 178 tissue specimens available for IHC analysis from patients who underwent surgical resection during 2008–2011 at Peking University Cancer Hospital & Institute were enrolled in this study. For IHC, each tissue specimen was formalin-fixed after resection, and then embedded with paraffin. All the included patients have histological diagnosis of GC, no neoadjuvant chemotherapy, and complete clinical records. The TNM stage of GC was classified according to the 8th edition of classification recommended by the American Joint Committee on Cancer (AJCC). The median follow-up duration of patients since the time of diagnosis was 39.96 months. In total, 85 patients died in the follow-up period. All patients provided informed consent for obtaining the tissue specimens. This study was approved by the Clinical Research Ethics Committee of Peking University Cancer Hospital & Institute, and all patients involved in this study provided written informed consent.

### Human HFn and magneto-HFn (M-HFn) protein biosynthesis and purification

The human HFn and M-HFn protein was synthesized according to the descriptions of our previous reports^12^. Detailed information about HFn and M-HFn biosynthesis and purification is described in the Supplementary materials.

### IHC and IF assays

The M-HFn nanoparticle-based IHC assay for GC tissues was performed as previously reported^12^. As negative controls, the sections were processed using the same protocol, except that they were not incubated with the M-HFn nanoparticles. The staining of TfR1 was examined and scored by two independent pathologists under microscopy, who were blind to the patient clinical data. Immunoreactivity score together with the staining intensity were used to assess the staining of TfR1. Finally, the staining levels of TfR1 expression were ascribed to − and +, low expression; ++ and +++, high expression.

For IHC, primary antibodies were used to detect anti-transferrin receptor antibody (anti-TfR1, Abcam, Cambridge, UK), PCNA (Dako, Carpinteria, CA, USA), and Ki67 antibody (Abcam, Cambridge, UK). For IF, primary antibodies were used to detect CD44 (Dako, Carpinteria, CA, USA) and 1B50-1 (anti-CACNA2D1), gifted by Prof. Zhang, Peking University, with visualization using fluorescein AF488 and AF594 (Jackson Alexa Fluor). DAPI was used to detect nuclei.

### Evaluation of antitumor activity in GC patient-derived xenograft (GC-PDX) mouse model

Detailed information about formation of HFn-Dox nanoparticles and their antitumor activity in the mouse model was described in the supplementary materials. GC-PDX mouse models were obtained from GC-PDX mouse bank in Peking University Beijing Cancer Hospital.

### Cell lines and FACS analysis

There were six GC cell lines (BGC823, SGC7901, AGS, HGC27, N87, and GES1) and one HEK293FT cell line used in this study. However, we focused on three cell lines for the further functional study: HGC27, SGC7901, and BGC823, which were obtained from Cell Research Institute, Shanghai, China. Cells were cultured in Dulbecco’s modified Eagle’s medium (Gibco BRL) supplemented with 10% fetal bovine serum (FBS, Gibco BRL) in a humidified atmosphere of 5% CO_2_ at 37 °C. Cell lines were authenticated using short-tandem repeat analysis and tested for mycoplasma contamination.

For FACS analysis, cells were dispersed, labeled, and analyzed as protocol-described HFn^12^. HFn was directly labeled with PE-Cy5 using Lightning-Link conjugation kit following the protocol (Innova Biosciences Ltd., Cambridge, UK).

### Tumorigenicity assay in NOD/SCID mice

For the tumorigenicity assay, 3 × 10^3^ and 3 × 10^2^ FACS TfR1-negative and -positive cells were suspended in 200 µl of a 1:1 mix of DMEM and Matrigel (BD Biosciences, Bedford, MA, USA), and transplanted subcutaneously into the back leg of nonobese diabetic/Prkdc severe combined immune-deficiency (NOD/SCID) mice with approximated body weights of 18 g or so (5-week old, Vitalriver, Beijing, China). Tumor formation was observed three times every week.

All the animal experiments followed the Animal Care Guidelines of Peking University Cancer Hospital & Institute. NOD/SCID mice were randomly assigned into four groups, and every group has three mice.

### Cell invasion assay

#### Boyden chamber invasion assay

The upper chamber was coated or not with 2 mg/ml Matrigel, and placed above the lower chamber with DMEM supplemented with 10% FBS. Then FACS TfR1-negative and -positive cells in serum-free DMEM (2 × 10^4^ for both cells) were added to the upper chamber (coated with Matrigel or not) or together with 1B50-1 antibody (which detected CACNA2D1, gifted by Prof. Zhang), which was given to these cells at the final concentration of 17 µg/ml. After 18 and 36 h, migration and invasion were performed in a 37 °C incubator. Three independent experiments were performed.

#### RTCA xCELLigence real-time cell proliferation/invasion assay

An optimal number of cells per well (proliferation: 1.5 × 10^3^ cells/well in complete medium; invasion: SGC7901 for 3 × 10^4^ and BGC803 for 4 × 10^4^ cells/well with serum-free medium in the upper chamber and complete medium in the below chamber) were seeded as an initial experiment in RTCA E-plate 16 or CIM-16 plates with 0.5 mg/ml Matrigel (RTCA; xCELLigence Roche, Penzberg, Germany). According to the xCELLigene manufacturer’s instruction, baseline was measured. Plates were incubated for 30 min at room temperature before starting the measurements. Cell index was measured in a time-resolved manner (every 10 min during 65 h for proliferation assay; every 1 h during 40 h for invasion assay). Three independent experiments were performed.

### TUNEL assay

A TUNEL assay-based in situ cell death detection kit (Roche Diagnostics GmbH, Mannheim, Germany) was used to detect the apoptotic cell death that had occurred in tumor grafts treated with HFn-Dox, Dox-free, and 1× phosphate-buffered saline (PBS), respectively, following the protocols recommended by the manufacturer. Slides were mounted in 90% glycerol/PBS after staining nuclei with DAPI. The slides were observed under a fluorescent confocal microscope (Leica, Wetzlar, Germany).

### 3D culturing

After sorting, TfR1-negative and -positive cells were plated on 24-well plates coated with Matrigel (250 µl/well, BD Biosciences) at a density of 200/well, and cultured for 2 weeks in DMEM medium supplemented with 10% FBS, with the medium being changed every 48 h. Three independent experiments were performed.

### RNA-seq analysis

Total RNA was isolated from sorted SGC7901 cells (TfR1 negative and positive) by flow cytometry analysis. RNA extraction was performed using RNeasy Micro Kit (Qiagen, Germany) following the manufacturer’s protocol. Total RNA was delivered to The Shanghai Biotechnology Corporation for sequencing with Illumina HiSeq2500. FPKM (fragments per kilobase of exon model per million mapped reads) was used to standardize the gene expression level. Statistics for differentially expressed genes were calculated by a *q* value ≤ 0.05 and fold change ≥ 2 (RNA-seq: GSE143497).

### LentiCRISPRv2-mediated sgRNA targeting TfR1 knockout

TfR1-knockout expression was established by CRISPR–Cas9 targeting TfR1 in BGC823 and SGC7901 cell lines. Lentivirus was produced by co-transfection of HEK293FT cells with LentiCRISPRv2, LentiCRISPRv2_sgTfR1-1, and LentiCRISPRv2_sgTfR1-2, respectively, together with lentiviral-packaging mix (Invitrogen, Carlsbad, CA, USA), according to the manufacturer’s instruction, which was transfected with Lipofectamine 2000 (Life Technologies). For the detailed sequences of the sgRNA: sgTfR1-1: 5′-CACCCG CTA TAC GCC ACA TAA CCC CC-3′; sgTfR1-2: 5′-CACCCG CTG CAG CAC GTC GCT TAT AT-3′. Stable TfR1-knockout cells were selected with puromycin (1.0 mg/ml) for 14 days after transfection for 48 h, and successfully TfR1-knockout cell clones were identified by flow cytometry.

### IFN-γ-responsive assay

An optimal number of cells (3 × 10^5^) were plated on a six-well plate for 12 h, then treated with IFN-γ (10 ng/ml) for 24 h, and extracted with the corresponding RNA to detect IFN-γ-inducible genes (*PD-L1*, *CXCL9*, and *CXCL10*), the primers of these genes are listed in Table S3. Three independent experiments were performed.

### Gene set enrichment analysis (GSEA)

Data used for GSEA were accessible from RNA-seq results and TCGA database (http://gdac.broadinstitute.org/), and analyzed by the software GSEA v3.0 (http://www.broadinstitute.org/gsea). The high and low groups of TCGA GC specimens were separated by the upper and lower 100 cases with TFRC expression level. False-discovery rate was set at 0.20.

### Statistics

Statistical analysis was carried out by SPSS20.0 software (SPSS Inc., Chicago, IL). Chi-square test or Fisher’s exact test was applied to assess the correlation between TfR1 expression and clinical parametric distribution in GC patients. The association of TfR1 expression with OS and PFS was analyzed by Kaplan–Meier curves and Log-rank test. Univariate and multivariate analysis were used to determine the potential prognostic factors with Cox regression model. Mann–Whitney *U* test or Student’s *t* test was performed to compare the significant cell function between two cell groups. The variance was similar between the statistically compared groups. All tests of statistical significance were two-sided; *P* < 0.05 was judged as the statistically significant level.

## Supplementary information

Supplemental

Supplemental

Figure s1

Figure s2

Figure s3
