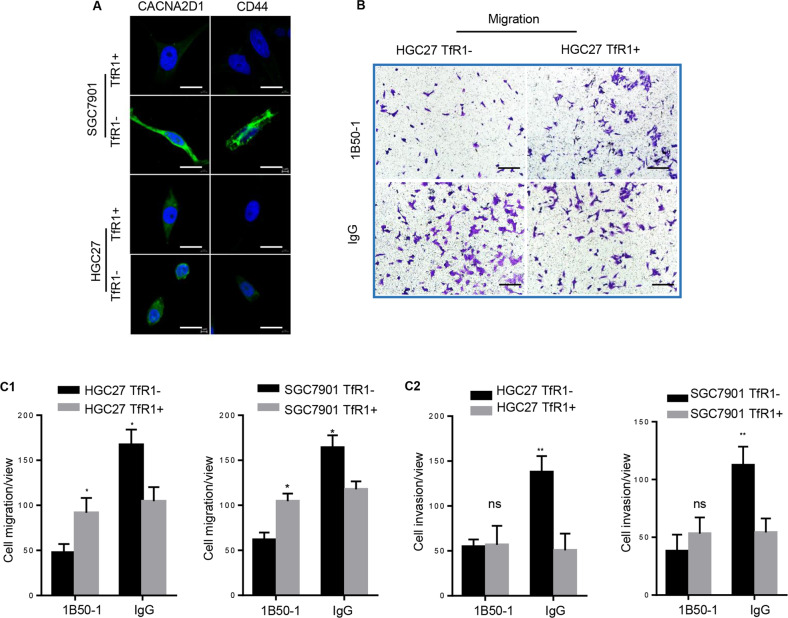# Correction: TfR1 binding with H-ferritin nanocarrier achieves prognostic diagnosis and enhances the therapeutic efficacy in clinical gastric cancer

**DOI:** 10.1038/s41419-022-05453-w

**Published:** 2022-11-25

**Authors:** Xiaojing Cheng, Kelong Fan, Lin Wang, Xiangji Ying, Andrew J. Sanders, Ting Guo, Xiaofang Xing, Meng Zhou, Hong Du, Ying Hu, Huirong Ding, Ziyu Li, Xianzi Wen, Wenguo Jiang, Xiyun Yan, Jiafu Ji

**Affiliations:** 1 Key Laboratory Carcinogenesis and Translational Research (Ministry of Education/Beijing), Division of Gastrointestinal Cancer Translational Research Laboratory, Peking University Cancer Hospital & Institute, Beijing, China; 2 Key Laboratory of Protein and Peptide Pharmaceutical, Chinese Academy of Sciences and University of Chinese Academy of Sciences, Beijing, China; 3 Key Laboratory Carcinogenesis and Translational Research (Ministry of Education/Beijing), Department of Gastrointestinal Surgery, Peking University Cancer Hospital & Institute, Beijing, China; 4 Department of Gastrointestinal Surgery, Beijing Shijitan Hospital, Capital Medical University, Beijing, China; 5 Cardiff China Medical Research Collaborative (CCMRC), Cardiff University School of Medicine, Heath Park, Cardiff, UK; 6 Key Laboratory Carcinogenesis and Translational Research (Ministry of Education/Beijing) Department of Biobank, Peking University Cancer Hospital & Institute, Beijing, China; 7 Key Laboratory Carcinogenesis and Translational Research (Ministry of Education/Beijing), Division of Central Laboratory, Peking University Cancer Hospital & Institute, Beijing, China

**Keywords:** Targeted therapies, Cancer stem cells

Correction to: *Cell Death and Disease* 10.1038/s41419-020-2272-z, published online 05 February 2020

The original version of this article contained an error. The authors put the wrong picture (upper right) in Fig. 4B. The authors apologize for the error. The corrected Figure can be found below.