# Supplementary material for: TfR1 binding with H-ferritin nanocarrier achieves prognostic diagnosis and enhances the therapeutic efficacy in clinical gastric cancer
Source: Cell Death Dis. 2020 Feb 5;11(2):92. doi: 10.1038/s41419-020-2272-z (PMC7002446; doi:10.1038/s41419-020-2272-z)
Supplement: Supplementary file 1 — Supplemental [file 41419_2020_2272_MOESM1_ESM.docx]

**Supplemental materials and methods:**

**Human HFn protein biosynthesis and purification**

The expression vector HFn-pET-30a (+) was transformed into *E. coli* BL21 (DE3) (TransGen Biotech, China) according to the manufacturer’s instructions. HFn protein was expressed in *E. coli* where it self-assembled into the 24 subunit nano-cage. The transformed *E. coli* cells were grown overnight in LB medium with 50 mg/L kanamycin. Then, HFn protein production was induced by isopropyl-β-D-thiogalactoside (IPTG, 0.75 mM, Sigma-Aldrich, Merk, USA), and cells were incubated for an additional 4 h. After the incubation, *E. coli* cells were harvested by centrifugation at 4,500 g for 45 min and the pellets were resuspended in TBS buffer (20 mM Tris, 150 mM NaCl, pH 8.0).

Then, the resuspended *E. coli* cells was sonicated on ice and centrifuged at 15,000 g for 30 min. The supernatant was heated at 72℃ for 10 min to denature and precipitate most *E. coli* proteins. After centrifugation, the HFn proteins in supernatant were precipitated by ammonium sulfate (520 g/L). The precipitate was collected by centrifugation at 22,000 g for 45 min, and then dissolved in PBS buffer (50 mM NaH_2_PO_4_, 150 mM NaCl, pH 7.4). After dialyzing out the ammonium sulfate, DNAse and RNAse (Sigma-Aldrich) were added to final concentration of 60 and 100 μg/mL, respectively, and incubated for 30 min at room temperature. Finally, HFn protein was purified by size exclusion chromatography on a Sepharose 6 PG XK 16/70 column (GE Healthcare, USA) followed by ion-exchange chromatography on Q-Sepharose Fast Flow (GE Healthcare, USA). The concentration of HFn was determined in triplicate by the BCA protein assay kit (Pierce, Thermo Scientific, and Rockford, USA) using bovine serum albumin as the standard. The typically yield of HFn was 100 mg per 1 L patch.

**Preparation of magneto-HFn (M-HFn) nanoparticle**

The degassed solution of HFn protein (0.25 mg/ml in 100 mM NaCl) was added to a jacketed reaction vessel under N_2_. The temperature of the reaction vessel was kept at 65℃ and the pH was titrated to 8.5. Ammonium iron (II) sulfate hexahydrate (NH_4_)_2_Fe (SO_4_)_2_·6H_2_O was added as an iron source at a rate of 100 Fe/ (protein•min) to attain a theoretical loading factor of 5000 Fe molecules per protein. Simultaneously, freshly prepared H_2_O_2_ was added at a stoichiometric equivalent of 1:3 H_2_O_2_:Fe^2+^ as an oxidant. The reaction was considered complete 5 min after the addition of iron and H_2_O_2_. Sodium citrate was added to chelate any free iron. The synthesized M-HFn nanoparticles were centrifuged to remove the aggregated nanoparticles and then dialyzed against phosphate-buffered saline (PBS) overnight. The concentration of M-HFn nanoparticles was assumed to be the same as that of HFn protein and was determined using a BCA protein assay kit (Pierce, ThermoFisher Scientific Inc, Waltham, USA). Purified M-HFn nanoparticles were obtained with a yield of >90%. We also determined that the iron loading of M-HFn was 1985.1 iron atoms per HFn protein shell. The concentration of HFn protein was quantified by BCA test, and the concentration of iron was determined by ICP-MS after acidolysis of M-HFn with aqua regia.

**Formation of the HFn-doxorubicin (HFn-Dox) nanoparticles**

HFn protein was added to 8 M urea (Sinopharm Chemical Reagent Co., Ltd, Shanghai, China) solution with the volume of 50 ml to reach a final protein concentration of 1 mg/mL. Then, the Dox (Sangon Biotech, Shanghai, China) was dissolved to the final concentration of 1 mg/mL in the solution. The mixed solution was incubated for 30 minutes at room temperature kept in the dark to ensure the complete dissociation of HFn protein. Then, the mixed solution was transferred to dialysis bags (Spectrum Labs) with the molecular weight cut-off (MWCO) of 3.5 kDa, and dialyzed against gradients of urea (7M-5M-3M-2M-1M, each for 4 hours, in dark) buffers at 4℃ to reassemble the protein shells of HFn. The resulting solution was finally dialyzed against PBS overnight to completely remove free Dox molecules outside of the protein shell. After that, the solution was centrifuged at 15,000 rpm for 30 min to remove the precipitates. The solutions of HFn-Dox nanoparticles were concentrated using Vivaspin-20 centrifugal filters (MWCO 100 kDa, Sartorius). Finally, the HFn-Dox nanoparticles were purified through size exclusion chromatography (Sepharose 6 PG XK 16/70 column, GE Healthcare, USA). The concentration of Dox was determined using the absorbance at 495nm and the Dox molar extinction coefficient of 1.00×10^4^ M^-1^ cm^-1^ by UV-Vis spectroscopy (Nanodrop 2000, ThermoFisher Scientific Inc, Waltham, USA). The concentration of HFn protein for HFn-Dox nanoparticle was determined in triplicate by the BCA protein assay kit (Pierce, ThermoFisher Scientific Inc, Waltham, USA) using bovine serum albumin as the standard and subtracted the absorption (in 562 nm) of same dose of free Dox. Purified HFn-Dox nanoparticles were obtained with a yield of about 95%, and the average molecules of doxorubicin per HFn molecule was approximately 36.

For the size exclusion chromatogram of HFn-Dox after purification and HFn-free. We can see from the chromatogram that a specific 485 nm absorption peak appears at the same position of the 280 nm absorption peak in the spectrum of HFn-Dox, which indicates that Dox had been successfully loaded into HFn. Moreover, the position of the 280 nm absorption peak of HFn-Dox is the same as that of HFn, which indicates Dox loading had little influence on the particle size of HFn (Fig. S2).

**Evaluation of antitumor activity in GC patient-derived xenograft (GC-PDX) mouse model*.***

TfR1 positive GC-PDX mouse models were randomly selected according the results of IHC performed on the original GC and Tumor Graft tissues using M-HFn nanoparticles. When the tumor volume reached 100 mm^3^, the mice were randomly assigned to treatment and control groups (n=5 mice in each group), and HFn-Dox (5 mg/kg body weight (BW), Dox equivalents), free-Dox (5 mg/kg BW) and free HFn protein (120 mg/kg BW, equivalent to 5 mg/kg HFn-Dox dose) was administrated intravenously to the forth-generation of mice on day 7, 12, 18, and 23, respectively. The volume of tumors and body weight of the mice was measured 2 times a week during the experimental period. Tumor volume was determined by caliper measurement with the formula V_tumor_ = *L* × *W*^2^/2, where *L* and *W* refer to the maximum and minimum diameters of tumor, respectively. The tumor bearing mice were monitored for up to 55 days after implantation, or until one of the following conditions for sacrificing them were met: (a) 15% body weight loss or (b) tumor greater than 1000 mm^3^. On day 55, all surviving mice were sacrificed and analyzed.

**H&E staining**

Formalin fixed paraffin embedded GC and Tumor Grafts from PDX models were dewaxed and rehydrated in xylem and ethanol. The sections were stained with hematoxylin and eosin to assess the morphology. The sections of Tumor Graft samples were examined by pathologists to exclude spontaneous lymphomas in the NOD/SCID mice.

**RNA extraction and qPCR analysis**

The total RNA was extracted using Trizol reagent (Invitrogen, Carlsbad, CA, USA) and cDNA synthesized by M-MLV reverse transcriptase (Promega, Madison, WI, USA) according to the manufacturer's instructions. Gene expression level was determined on ABI 7500 Fast instrument using Power SYBR Green Master Mix (ThermoFisher Scientific Inc, Waltham, USA). Each sample was tested in triplicate, ∆∆CT method was used to analyze the fold change in the gene expression level normalized by *GAPDH*. The primer sequences of *Sox2*, *Nanog*, *Oct4* and *CACNA2D1* were cited from Zhao et al^1^, and other primer sequences were listed in Table S3.

**Colony formation assay**

For the clonogenic assay, 500 cells were inoculated into 6-well plate and cultured for 14 days, the medium was changed one time during these days. Fixed for 30minutes with 4% paraformaldehyde and stained with 0.5% crystal violet for 10 minutes. Photos were taken with a digital camera and colony counting was performed. All experiments were conducted three times in triplicates.

**Western blotting**

Cells were lysed in pre-chilled RIPA lysis buffer (Merk, USA) containing protease inhibitor cocktail and phosphatase inhibitor cocktail (Selleck chemicals, CA, USA). Proteins were separated by different concentration of SDS-PAGE gels depended on the molecular weight of target protein, and transferred to 0.22μm PVDF membrane. After blocking, the membranes were incubated with primary antibodies, then incubated with HRP-conjugated secondary antibodies and developed by the enhanced Chemiluminescence Detection System (GE Healthcare, Chicago, IL, USA). Primary antibodies were listed as follows: E-cadherin (CST,#3195), N-cadherin (CST,#4061), Snail (CST,#3879), Stat3 (CST,#12640), P-Stat3 (CST,#52075), PI3K (CST,#4249), Akt (CST,#4685), P-Akt (CST,#4060) and GAPDH (CST, #5174).

**Reference**:

1. Zhao, W.*, et al.* 1B50-1, a mAb raised against recurrent tumor cells, targets liver tumor-initiating cells by binding to the calcium channel alpha2delta1 subunit. *Cancer Cell* **23**, 541-556 (2013).
