# Supplementary material for: TfR1 binding with H-ferritin nanocarrier achieves prognostic diagnosis and enhances the therapeutic efficacy in clinical gastric cancer
Source: Cell Death Dis. 2020 Feb 5;11(2):92. doi: 10.1038/s41419-020-2272-z (PMC7002446; doi:10.1038/s41419-020-2272-z)
Supplement: Supplementary file 2 — Supplemental [file 41419_2020_2272_MOESM2_ESM.docx]

**Supplemental tables:**

**Table S1 Correlation of TfR1 expression with clinicopathological features in gastric cancer patients**

| Variables |  | Case No. | TfR1 expression | |  | *P* value |
| --- | --- | --- | --- | --- | --- | --- |
|  |  |  | Low | High |  |  |
| Age |  |  |  |  |  | 0.694 |
| ≤60 |  | 95 | 44 | 51 |  |  |
| >60 |  | 83 | 36 | 47 |  |  |
| Gender |  |  |  |  |  | 0.222 |
| Male |  | 116 | 56 | 60 |  |  |
| Female |  | 62 | 24 | 38 |  |  |
| Lymph node metastasis |  |  |  |  |  | 0.353 |
| N0 |  | 53 | 21 | 32 |  |  |
| N1+2+3 |  | 125 | 59 | 66 |  |  |
| Depth of invasion |  |  |  |  |  |  |
| T1+2 |  | 34 | 13 | 21 |  | 0.404 |
| T2+3 |  | 143 | 66 | 77 |  |  |
| Undetermined |  | 1 | 1 | 0 |  |  |
| Distant metastasis |  |  |  |  |  | 0.347 |
| Mo |  | 161 | 74 | 87 |  |  |
| M1 |  | 15 | 5 | 10 |  |  |
| Undetermined |  | 2 | 1 | 1 |  |  |
| Differentiation |  |  |  |  |  |  |
| Poor |  | 86 | 38 | 48 |  | 0.986 |
| Moderate+Well |  | 88 | 39 | 49 |  |  |
| Undetermined |  | 4 | 3 | 1 |  |  |
| Gross type |  |  |  |  |  | 0.429 |
| Ulcerative type |  | 154 | 67 | 87 |  |  |
| Protrude type |  | 7 | 3 | 4 |  |  |
| Others |  | 11 | 7 | 4 |  |  |
| Undetermined |  | 6 | 3 | 3 |  |  |
| Tumor size |  |  |  |  |  | 0.247 |
| ≤5.0cm |  | 97 | 40 | 57 |  |  |
| >5.0cm |  | 79 | 39 | 39 |  |  |
| Undetermined |  | 3 | 1 | 2 |  |  |
| Location |  |  |  |  |  | **0.010** |
| Proximal |  | 48 | 29 | 19 |  |  |
| Distal |  | 129 | 50 | 79 |  |  |
| Undetermined |  | 1 | 1 | 0 |  |  |
| Lauren |  |  |  |  |  | 0.451 |
| Diffuse |  | 42 | 22 | 20 |  |  |
| Intense |  | 98 | 40 | 58 |  |  |
| Mixed |  | 36 | 16 | 20 |  |  |
| Undetermined |  | 2 | 2 | 0 |  |  |
| TNM stage |  |  |  |  |  | 0.245 |
| I+II |  | 78 | 31 | 47 |  |  |
| III+IV |  | 99 | 48 | 51 |  |  |
| Undetermined |  | 1 | 1 | 0 |  |  |

Chi-square test and Fisher exact test; Proximal: cardiac and gastroesophageal junction; Distal: gastric.

**Table S2:** **The dysregulated genes in the RNAseq analysis.**

| Gene Symbol | Gene ID | Full Name | log2FC | Adjust *P* value |
| --- | --- | --- | --- | --- |
| Upregulation |  |  |  |  |
| KLF4 | ENSG00000136826 | Kruppel-like factor 4 | 1.42 | 7.68E-07 |
| PAX6 | ENSG00000007372 | paired box 6 | 4.16 | 2.68E-07 |
| ISL1 | ENSG00000016082 | ISL LIM homeobox 1 | 1.51 | 0.011102633 |
| **IFIT2** | **ENSG00000119922** | **interferon induced protein with tetratricopeptide repeats 2** | **3.04** | **3.33E-34** |
| **FOLR1** | **ENSG00000110195** | **folate receptor 1** | **3.73** | **2.87E-46** |
| **MBNL3** | **ENSG00000076770** | **muscleblind-like splicing regulator 3** | **4.73** | **8.58E-40** |
| **LRRK1** | **ENSG00000154237** | **leucine-rich repeat kinase 1** | **4.11** | **1.91E-35** |
| **HERC5** | **ENSG00000138646** | **HECT and RLD domain containing E3 ubiquitin protein ligase 5** | **3.89** | **2.18E-45** |
| **OASL** | **ENSG00000135114** | **2'-5'-oligoadenylate synthetase-like** | **3.43** | **2.61E-42** |
| **IFIH1** | **ENSG00000115267** | **interferon induced, with helicase C domain 1** | **3.61** | **3.69E-36** |
| **OAS3** | **ENSG00000111331** | **2'-5'-oligoadenylate synthetase 3** | **3.27** | **3.40E-36** |
| FZD3 | ENSG00000104290 | frizzled class receptor 3 | 3.00 | 3.29E-08 |
| FZD10 | ENSG00000111432 | frizzled class receptor 10 | 4.46 | 4.63E-22 |
| BMP2 | ENSG00000125845 | bone morphogenetic protein 2 | 2.66 | 1.07E-23 |
| TBX3 | ENSG00000135111 | T-box 3 | 1.14 | 0.000129473 |
| ABCA13 | ENSG00000179869 | ATP binding cassette subfamily A member 13 | 2.53 | 4.07E-05 |
| ABCG2 | ENSG00000118777 | ATP binding cassette subfamily G member 2 | 1.27 | 9.15E-05 |
| ABCA1 | ENSG00000165029 | ATP binding cassette subfamily A member 1 | 1.54 | 0.008931622 |
| JAK2 | ENSG00000096968 | Janus kinase 2 | 1.21 | 0.000979348 |
| ITGB4 | ENSG00000132470 | integrin subunit beta 4 | 1.98 | 3.03E-14 |
| IL7R | ENSG00000168685 | interleukin 7 receptor | 1.09 | 0.000881845 |
| FGF2 | ENSG00000138685 | fibroblast growth factor 2 | 1.09 | 0.000257698 |
| STAT5A | ENSG00000126561 | signal transducer and activator of transcription 5A | 2.17 | 7.56E-09 |
| IL15 | ENSG00000164136 | interleukin 15 | 1.69 | 6.04E-07 |
| Downregulation | |  |  |  |
| **AKR1C2** | **ENSG00000151632** | **aldo-keto reductase family 1, member C2** | **-2.82** | **1.32E-30** |
| **AKR1C1** | **ENSG00000187134** | **aldo-keto reductase family 1, member C1** | **-2.71** | **9.70E-29** |
| **TUB** | **ENSG00000166402** | **tubby bipartite transcription factor** | **-3.73** | **1.25E-24** |
| PIK3R1 | ENSG00000145675 | phosphoinositide-3-kinase regulatory subunit 1 | -1.10 | 1.34E-05 |
| EFNA5 | ENSG00000184349 | ephrin-A5 | -1.12 | 3.38E-05 |
| SPP1 | ENSG00000118785 | secreted phosphoprotein 1 | -1.52 | 0.039587557 |
| ANGPT1 | ENSG00000154188 | angiopoietin 1 | -1.17 | 0.010933092 |
| CNTF | ENSG00000242689 | ciliary neurotrophic factor | -1.16 | 0.01044513 |
| FHL1 | ENSG00000022267 | four and a half LIM domains 1 | -1.07 | 6.53E-07 |

Abbreviations: N, sorted negative cells; P, sorted positive cells; FC, fold change.

**Table S3:** **Primer sequences used in this study.**

| Gene Name | Sequence (5'--3') |
| --- | --- |
| *CXCL9*-F | TCTTTTCCTCTTGGGCATCATC |
| *CXCL9*-R | CTTGGTTGGTGCTGATGCAG |
|  |  |
| *CXCL10*-F | TCTGAGCCTACAGCAGAGGAAC |
| *CXCL10*-R | GCTGATGCAGGTACAGCGTAC |
|  |  |
| *PDL1*-F | GCTGCACTAATTGTCTATTGGGA |
| *PDL1*-R | AATTCGCTTGTAGTCGGCACC |
|  |  |
| *GAPDH*-F | GAAGGTGAAGGTCGGAGT |
| *GAPDH*-R | GAAGATGGTGATGGGATTTC |

**Figure legends:**

**Fig. S1** **Comparing the M-HFn and anti-TfR1 staining in gastric cancer tissues**. **a-b** Positive staining for M-HFn particles (a) and anti-TfR1 antibody (b, brown). **c-d** Negative staining for M-HFn particles (c) and anti-TfR1 antibody (d). Scale bars, (Left) 100 µm; (Right) 50 µm.

**Fig. S2** **The size exclusion chromatogram of HFn-Dox and unloaded HFn**. **a-b** The spectrums were obtained by passing 2 mL of 3 mg/mL HFn-Dox (a) or HFn (b) through a Hiload 16/600 Superdex 200pg column. **c** The signals of HFn and Dox were obtained using AKTA Avant protein purification system by detecting the absorption peaks of the effluent at 280 nm and 485 nm, respectively.

**Fig. S3** **Combining TfR1- and CD44+ as a poor prognostic marker for GC patients, and analysis of TfR1 related genome features in TCGA datasets.** **a** Kaplan-Meier survival curves 5-year OS (left) and PFS (right) in GC patients with different CD44 protein expression (**a1**); prognostic value of combining TfR1 and CD44 expression levels was analyzed by Kaplan-Meier survival analysis (**a2**). **b** Hallmark gene sets enriched for commonly low (**b1**) and high (**b2**) *TFRC* group based on TCGA datasets, respectively. **c** Analysis of *TfR1* mRNA level correlated with *CD8A* (**c1**) and *CD8B* (**c2**) mRNA level in TCGA gastric cancer. **d** Correlation of *TfR1* expression level with stomach cancer patient survival depending on *CD8* [(*CD8A*+*CD8B*)/2] expression level of patient tumor. OS, overall survival; PFS, progression free survival.
