# Supplementary figures and images for: TfR1 binding with H-ferritin nanocarrier achieves prognostic diagnosis and enhances the therapeutic efficacy in clinical gastric cancer
Source: Cell Death Dis. 2020 Feb 5;11(2):92. doi: 10.1038/s41419-020-2272-z (PMC7002446; doi:10.1038/s41419-020-2272-z)

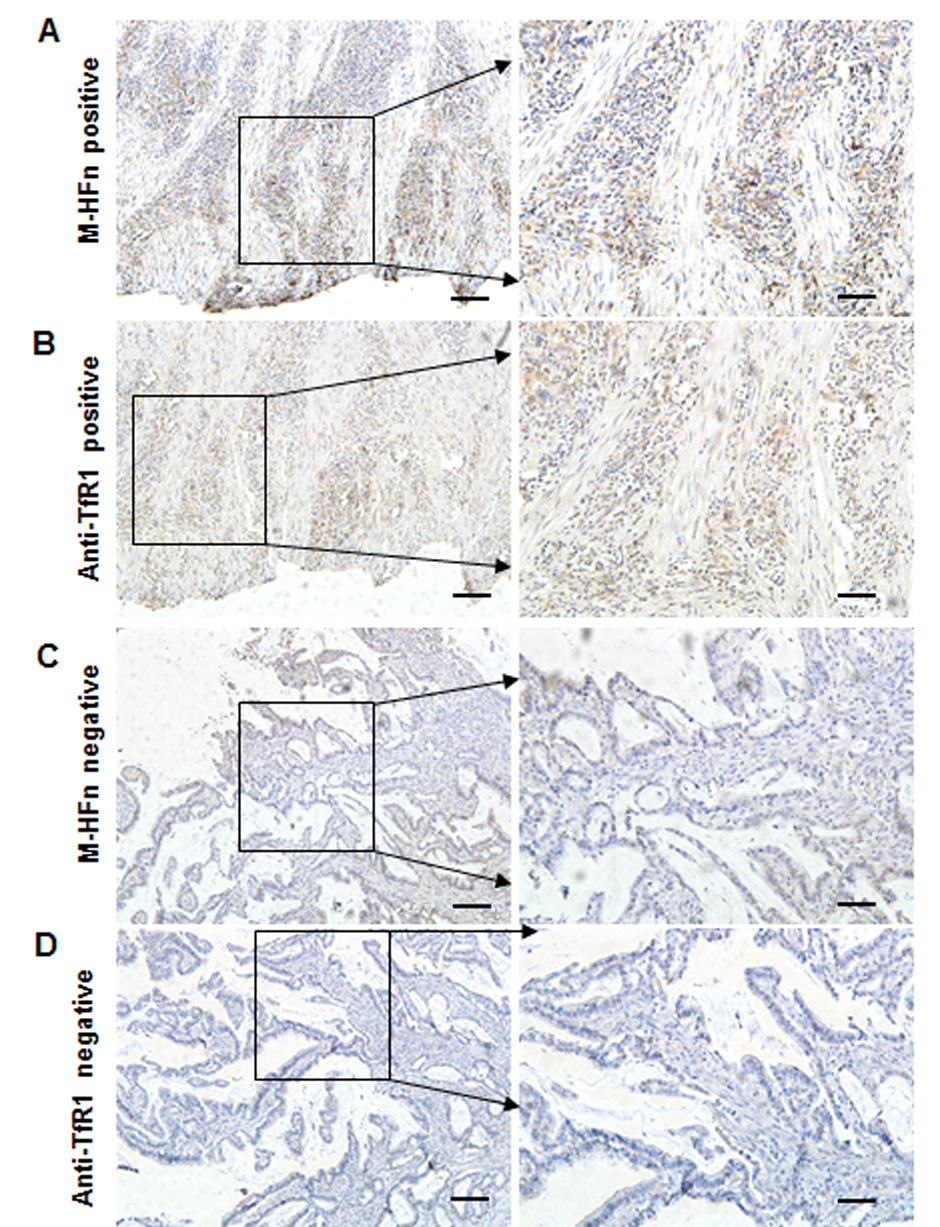

Supplement: Supplementary file 3 — Figure s1 [file 41419_2020_2272_MOESM3_ESM.tif]

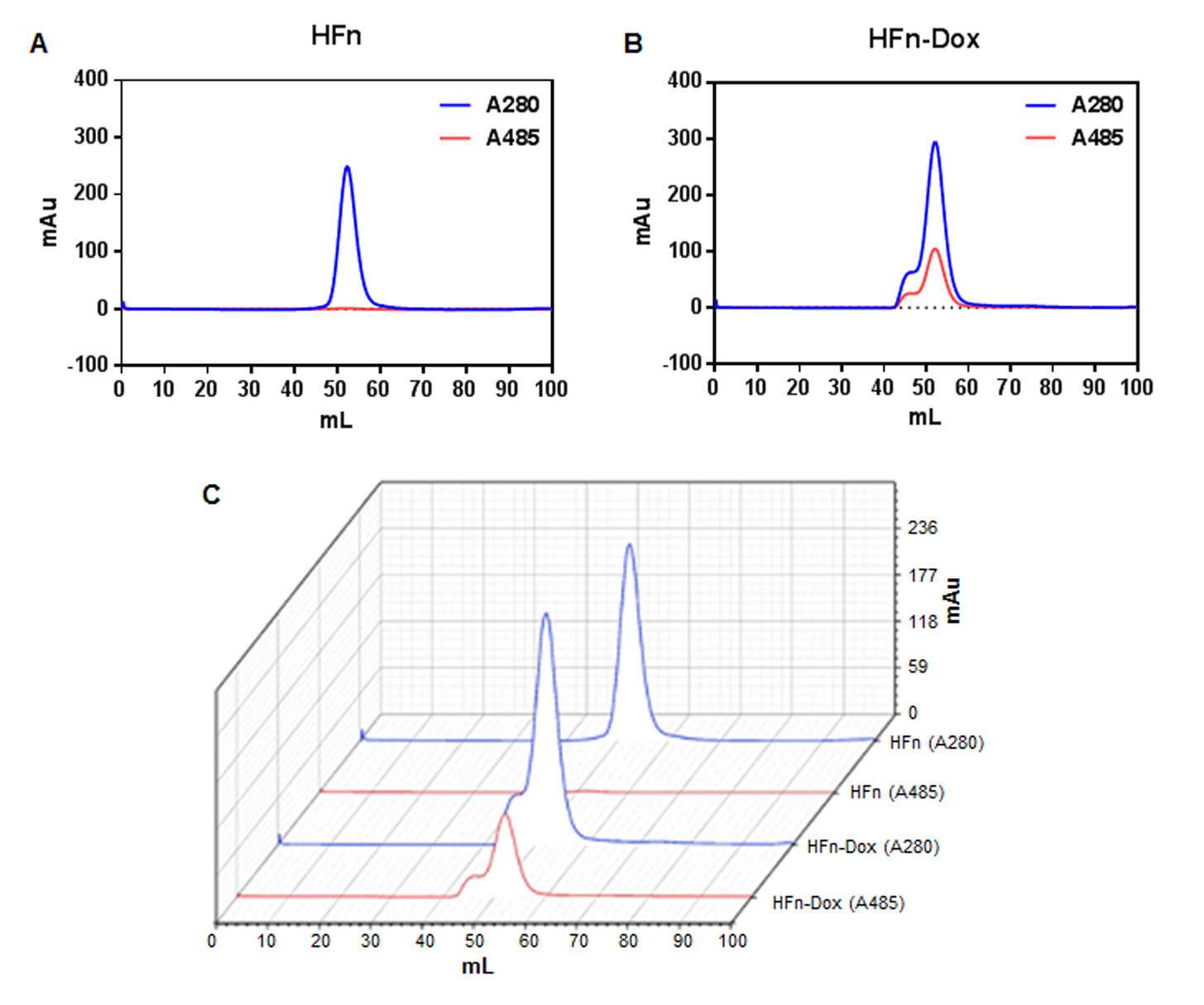

Supplement: Supplementary file 4 — Figure s2 [file 41419_2020_2272_MOESM4_ESM.tif]

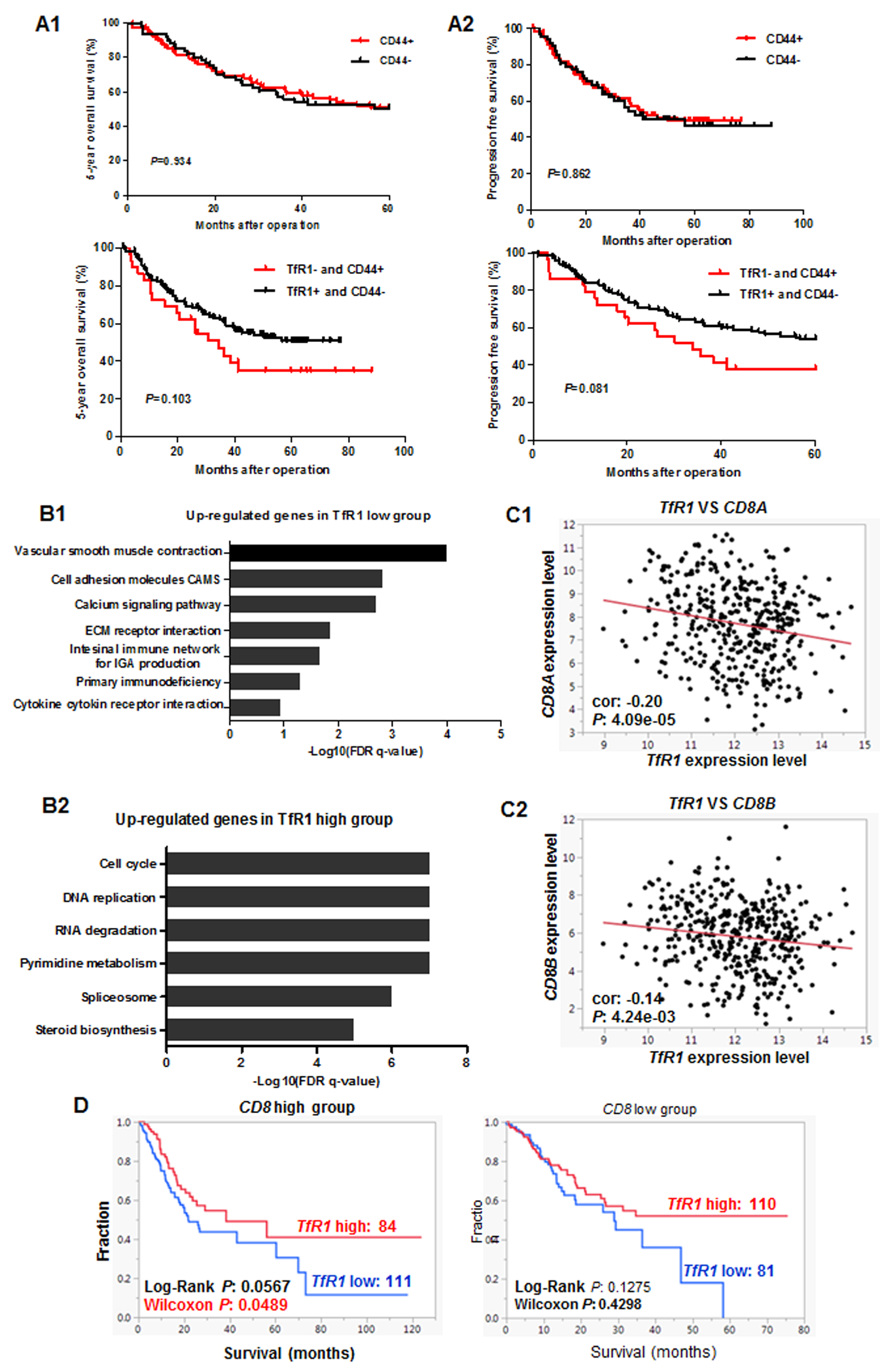

Supplement: Supplementary file 5 — Figure s3 [file 41419_2020_2272_MOESM5_ESM.tif]
